# Molecular Signatures of Recurrent Hepatocellular Carcinoma Secondary to Hepatitis C Virus following Liver Transplantation

**DOI:** 10.1155/2013/878297

**Published:** 2013-11-26

**Authors:** Trina Das, Deborah L. Diamond, Matthew Yeh, Sajida Hassan, Janine T. Bryan, Jorge D. Reyes, James D. Perkins

**Affiliations:** ^1^Division of Transplantation, Department of Surgery, University of Washington School of Medicine, Seattle, WA 98195-6410, USA; ^2^Department of Pharmaceutics, University of Washington, Seattle, WA 98121-1023, USA; ^3^Department of Laboratory Medicine, University of Washington Medical Center, Seattle, WA 98104-2499, USA; ^4^Department of Microbiology, University of Washington, Seattle, WA 98195-7735, USA

## Abstract

Chronic hepatitis C virus (HCV) induced hepatocellular carcinoma (HCC) is a primary indication for liver transplantation (LT). In western countries, the estimated rate of HCC recurrence following LT is between 15% and 20% and is a major cause of mortality. Currently, there is no standard method to treat patients who are at high risk for HCC recurrence. The aim of this study was to investigate the molecular signatures underlying HCC recurrence that may lead to future studies on gene regulation contributing to new therapeutic options. Two groups of patients were selected, one including patients with HCV who developed HCC recurrence (HCC-R) ≤3 years from LT and the second group including patients with HCV who did not have recurrent HCC (HCC-NR). Microarray analysis containing more than 29,000 known genes was performed on formalin-fixed-paraffin-embedded (FFPE) liver tissue from explanted livers. Gene expression profiling revealed 194 differentially regulated genes between the two groups. These genes belonged to cellular networks including cell cycle G1/S checkpoint regulators, RAN signaling, chronic myeloid leukemia signaling, molecular mechanisms of cancer, FXR/RXR activation and hepatic cholestasis. A subset of molecular signatures associated with HCC recurrence was found. The expression levels of these genes were validated by quantitative PCR analysis.

## 1. Introduction

Hepatitis C virus (HCV) infection is the most common cause of hepatocellular carcinoma (HCC) in the USA, Europe, and Japan, accounting for 47%–49%, 56%, and 75% of cases, respectively [1, 2]. HCC causes >600,000 deaths annually worldwide and is the most common primary liver cancer [3]. Definitive treatment for HCC is surgical resection when possible or liver transplantation for patients with end-stage liver disease and liver tumors. In USA, transplant guidelines stipulate that eligibility for liver transplantation is determined by the patient's liver tumor(s) meeting the Milan criteria (a single tumor ≤5 cm in diameter or up to 3 tumors with individual diameters ≤3 cm and no macrovascular invasion) [4]. Unfortunately, the recurrence of HCC is a major cause of mortality in surgically treated patients [5]. There is no standard therapy for patients who are at high risk for HCC recurrence. Hence, a better understanding of the molecular mechanisms involved in the recurrence of HCC post LT is necessary to develop an efficient surveillance protocol and seek new potential therapies.

Gene expression profiling is best performed on fresh or frozen tissue to lessen the degradation of RNA. However, collecting and storing this tissue are burdensome and costly, and obtaining Institutional Review Board (IRB) approval for longitudinal studies can be unsuccessful. Sample sizes are typically small, and collection requires years of time. In contrast, all clinical pathology laboratories have huge storage files containing formalin-fixed paraffin-embedded (FFPE) tissue. Formalin fixation has been used for several decades for preserving tissue. FFPE tissue would supply a huge resource for genomic studies to utilize previously collected samples with long-term clinical records. However, the formalin and storage can negatively influence the integrity of RNA extracted from the FFPE tissues [6]. RNA derived from FFPE specimens is partially degraded, resulting from the variability in tissue handling/processing, tissue sources, and RNA extraction methods [7]. However, advanced RNA isolation techniques have been developed [8]. Likewise, advanced technologies using a range of microarray platforms allow improved microarray gene expression profiling using FFPE tissues [9, 10]. This present study uses the advanced technologies of the complementary DNA-mediated annealing, selection, extension, and ligation (DASL) assay (Illumina, Inc., San Diego, CA, USA) to analyze FFPE stored liver tissue to look for potential molecular signatures of recurrent HCC.

Several gene-profiling studies have identified molecular signatures associated with HCC metastatic potential [11–13]. However, few common genes were identified in these studies [11–13]. This may have been due to the use of differing microarrays and gene selection procedures (i.e., algorithms), types of tissue sample studied, patients' clinical variability, the use of HCCs of different viral origins, that is, hepatitis B or C virus (HBV or HCV), which are major causes of human HCC, and to the small number of specimens studied [14, 15]. Most of these studies were performed on biopsy liver tissue samples. Hoshida et al. demonstrated the feasibility of genome-wide expression profiling of FFPE tissues using the DASL assay only for 6000 transcriptionally informative genes [16, 17]. This present feasibility study uses the method of transcriptome profiling of FFPE tissues to analyze 29,000 transcripts (DASL assay) for the clinical outcome of molecular signatures of HCC recurrence secondary to HCV chronic infection.

To help control for the variability encountered in other genomic studies, the present study performed microarray analysis utilizing FFPE stored liver tissue among a select group of patients, mostly male Caucasians with an average age of 55 years. Genes were successfully identified that are differentially expressed in tissue from post-LT patients with tumor recurrence and HCV compared with tissue from patients with no tumor recurrence and HCV after LT. This project has provided a subset of potential molecular signatures for future studies to explore their therapeutic and possible early marker potential for patients with HCC-R. This feasibility study has contributed information towards the better utilization of FFPE stored tissue and future large-scale multicenter expression analysis studies.

## 2. Methods 

### 2.1. Patient Population

After IRB approval from the University of Washington, all patients receiving liver transplants in the University of Washington program from January 1, 2000 until July 1, 2007 were retrospectively screened. Each patient was categorized for the presence of HCC in the explanted liver. All patients with HCC were followed until death or for at least 3 years until July 1, 2010. Recipient factors recorded included age, gender, race, body mass index (BMI), etiology of liver disease, pretransplant alpha-fetoprotein (AFP) level, any prior treatment for HCV, any treatment for HCC prior to transplantation, date of HCC recurrence after liver transplantation, and date of death. Tumor characteristics recorded at the time of explant included HCC histology, size of largest tumor, number of tumors, presence of tumor in both lobes of liver, presence of macroinvasion of HCC, and calculated predicting cancer recurrence score (PCRS) [5]. Two groups, patients with HCC recurrence and patients without HCC recurrence following liver transplantation, were identified and matched for the presence of HCV and the same gender for further genomic study.

### 2.2. Tissue Preparation

All explanted livers were stored in refrigerators at 4°C until being sliced into 1 cm sections to evaluate for the presence of HCC. HCC tissue was fixed in formalin and embedded in paraffin. Any characteristic tumor nodule was stained for diagnosis. In preparation for RNA extraction, the FFPE serial sections were cut into sections with a thickness of 10 *μ*m. The time period from storing the FFPE fixed tissue until preparation for RNA extraction was recorded. To differentiate nontumor tissue (adjacent cirrhotic tissue) and tumor tissue, hematoxylin and eosin (H+E) stained slides were microscopically evaluated by a pathologist. The tumor tissue was dissected from the nontumor tissue and sent for RNA extraction.

### 2.3. RNA Extraction, Purification, and Quality Assessment

Following deparaffinization, the tumor tissue was scraped from the slides into tubes and the RNA extracted using a modified protocol based on the Qiagen RNeasy FFPE kit (Qiagen, Valencia, CA, USA). Agilent 2100 bioanalysis (Agilent Technologies, Santa Clara, CA, USA) was performed to view the RNA quality. The cDNA synthesis was performed using the Qiagen Quantitect Reverse Transcription kit (Qiagen SpA, Milano, Italy). TaqMan quantitative polymerase chain reaction (qPCR) for the RPL-13A gene was performed using 200 ng of input RNA and 40 cycles of amplification as a quality assessment of the RNA; this is the RNA level quality control (QC) standard optimized by Illumina [16]. Similar studies by Abramovitiz [18] and Ton [19] had shown that a threshold *C*_*t*_ value of ≤29 would signify sufficient RNA quality to give reproducible results in the cDNA-mediated annealing, selection, extension, and ligation (DASL) assay; this threshold was used in our study.

### 2.4. Gene Expression Profiling

The whole genome DASL assay developed by Illumina is highly specialized to detect 1.3- to 2-fold changes in intact and partially degraded RNA from FFPE samples [10]. As previously described and validated by Hoshida et al., DASL assay (6000 genes) allows highly reproducible expression profiles with RNA derived from FFPE samples from HCC resection specimens [17]. The Illumina WG-DASL HumanRef-12_V4 Expression BeadChip microarrays, which quantify approximately 29000 transcripts, were used for our study. In the DASL assay, total RNA was first converted into cDNA in a reverse transcription reaction using biotinylated primers and resulted in fluorescent-labeled PCR products that were annealed to the BeadChips. The fluorescence of hybridized transcripts was captured by laser confocal microscopy scanning using the Illumina BeadArray Reader system [20]. The DASL assay was performed by the Fred Hutchinson Cancer Research Center (FHCRC) Genomics Resource.

### 2.5. Data Analysis

The raw DASL array data was exported via genomeStudio version 1.0 (Illumina, Inc.) to JMP Genomics (SAS Institute, Inc., Cary, NC, USA) for quantile normalization, principal component analysis (PCA), and log transformation. The data analysis QC standard was performed on the sample and chip level to ensure uniformly high quality microarray data. Initially, the quality of each BeadArray was assessed by examining the percent present calls (defined as the percent of bead types having a detection call *P* value <0.05), as well as plots of signal intensities for housekeeping, cy3_hyb, low_stringency_hyb, labeling, and biotin control bead types. In addition, log 2 transformation was performed, followed by quantile normalization and variance stabilizing transformation. For differential expression analyses, we followed the quintile with the greatest variance as previously described by Mittempergher and colleagues [21]. Genes differentially expressed between HCC-R and HCC-NR tumors were selected based on significance criteria of a false discovery rate less than 0.05 using analysis of variance (ANOVA) (≤0.05, with a fold change of 2). Heat maps were generated based on gene expression patterns through *k*-means clustering and viewed using CLUSTER 3.0 and TreeView 1.45 (software at http://eisenlab.org/), respectively. The threshold applied to our statistical tests was influenced by the large patient-to-patient variability in gene expression, although heterogeneity in expression is expected in human samples. Survival analysis was conducted using Kaplan-Meier curves and compared using the log-rank test.

### 2.6. Functional Data Analysis

Network analysis of the differentially expressed genes was performed using Ingenuity Pathway Analysis software (IPA) (Ingenuity Systems, Inc., Redwood City, CA, USA). The top canonical pathways that represent differentially regulated genes in the tumor tissues from the HCC recurrence group were evaluated. The top-scoring network of interactions is presented for the concurrent downregulated and the concurrent upregulated gene sets. This software analyzes molecular data in the context of known biological response and regulatory networks as well as other higher-order response pathways. Ingenuity functional analysis identified biological functions and/or diseases that were most significantly enriched and generated *P* values to determine the probability that each biological function assigned to that data set was due to chance alone.

### 2.7. Quantitative PCR (qPCR) Analysis

Quantitative PCR was performed on selected genes. The mRNA expression levels were quantified using a ViiA 7 real-time PCR System (Applied Biosystems, Life Technologies Corporation, Carlsbad, CA, USA) according to the manufacturer's instructions. In brief, total RNA was extracted from tumor tissues. First-strand cDNA synthesis was performed using the Quantitect Reverse Transcription kit (Qiagen SpA, Milano, Italy). Quantitative PCR for respective custom primer-probe sets (Applied Biosystems) was performed using 9.0 ng of input RNA and 40 cycles of amplification using the ViiA 7 real-time PCR system (Applied Biosystems). RPL-13A was used as the endogenous control for mRNA levels. Each experiment was run in duplicate, including RPL-13A as the endogenous control and repeated 3 times. Relative quantification was performed using the delta *C*_*t*_ method relative to RPL-13A as an internal control. The *P* values were calculated with Student's *t*-test.

## 3. Results

### 3.1. Clinicopathological Characteristics of the Patient Population

**Figure 1:**
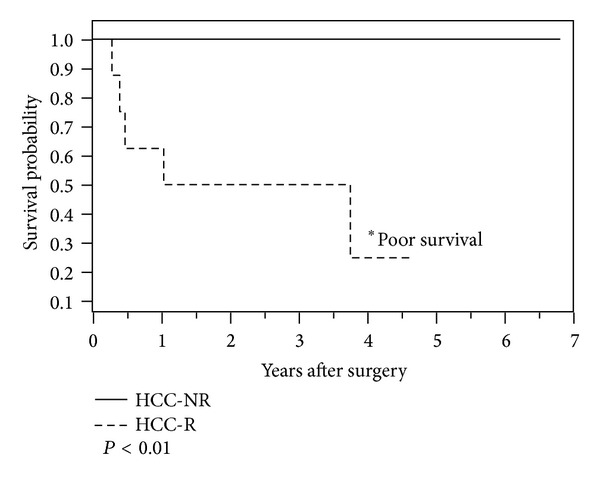
Kaplan-Meier survival curves comparing the high mortality rate in the tumor recurrence group with the mortality rate in the nonrecurrence group.

Of the 768 patients undergoing transplantation at the University of Washington from January 1, 2000 until July 1, 2007, 93 patients (12%) had HCC due to HCV. Of these, 11 male patients developed recurrent tumors within 3 years following transplantation. From this group, 10 male patients with recurrence (HCC-R) were matched with second cohort group of 20 patients in whom HCC did not recur (HCC-NR) for HCV status, age and BMI. Following review of the quality control (QC) metrics (see Section 3.2), only 8 patients from each group were considered for further study. The clinical characteristics of the HCC-R group (*n* = 8) and the HCC-NR (*n* = 8) group revealed all patients to be male, and most of them were Caucasian. There were no significant differences between the two groups with respect to age, BMI, prior HCV or HCC treatment, number of tumors, and presence of tumors in both lobes (Table 1). The HCC-R group showed a higher AFP level, (2268 ± 2837 mg/dL; *P* = 0.07) when compared with the HCC-NR group (138 ± 280 mg/dL). Likewise, the presence of macroinvasion and the PCRS was trending to be higher in the HCC recurrence group. The presence of poorly differentiated tumor was significantly higher (*P* < 0.01) in the HCC-R group (75%) versus the HCC-NR group (0%). Kaplan-Meier survival curves revealed that the HCC-R group had significantly (*P* < 0.01) lower 3-year survival (50%) compared with the HCC-NR group (100%) (Figure 1).

### 3.2. Quality Control Measure

**Figure 2:**
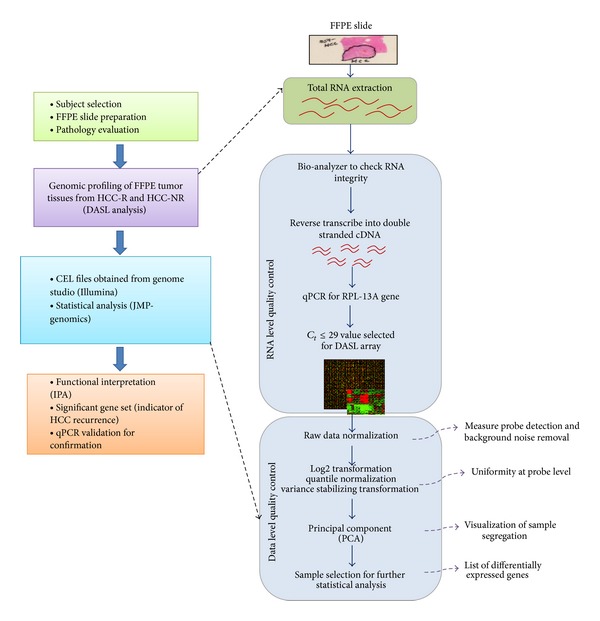
Experimental strategy for comparative transcriptome analysis of HCV-associated HCC with or without recurrence, using FFPE tissue to determine molecular signatures of HCC recurrence.

The present genomic study began with a sample size of 30 (HCC-R (*n* = 10) and HCC-NR (*n* = 20)). Following performance of QC metrics, 29 samples out of 30 (HCC-R 10/10 and HCC-NR 19/20) passed RNA level QC (*C*_*t*_ ≤ 29) and met the criteria for the WG-DASL array. Of these 29 samples following performance of the WG-DASL array, 16 (55%) samples (HCC-R: 8/10 (80%) and HCC-NR: 8/20 (40%)) met the data analysis QC standard and were selected for further comparative gene expression analysis (Figure 2). Overall, 14 (47%) of the samples were excluded from the final analysis.

### 3.3. Transcriptome Analysis Defines Differentially Regulated Genes, and Major Canonical Pathways Are Associated with Recurrence of HCC

**Figure 3:**
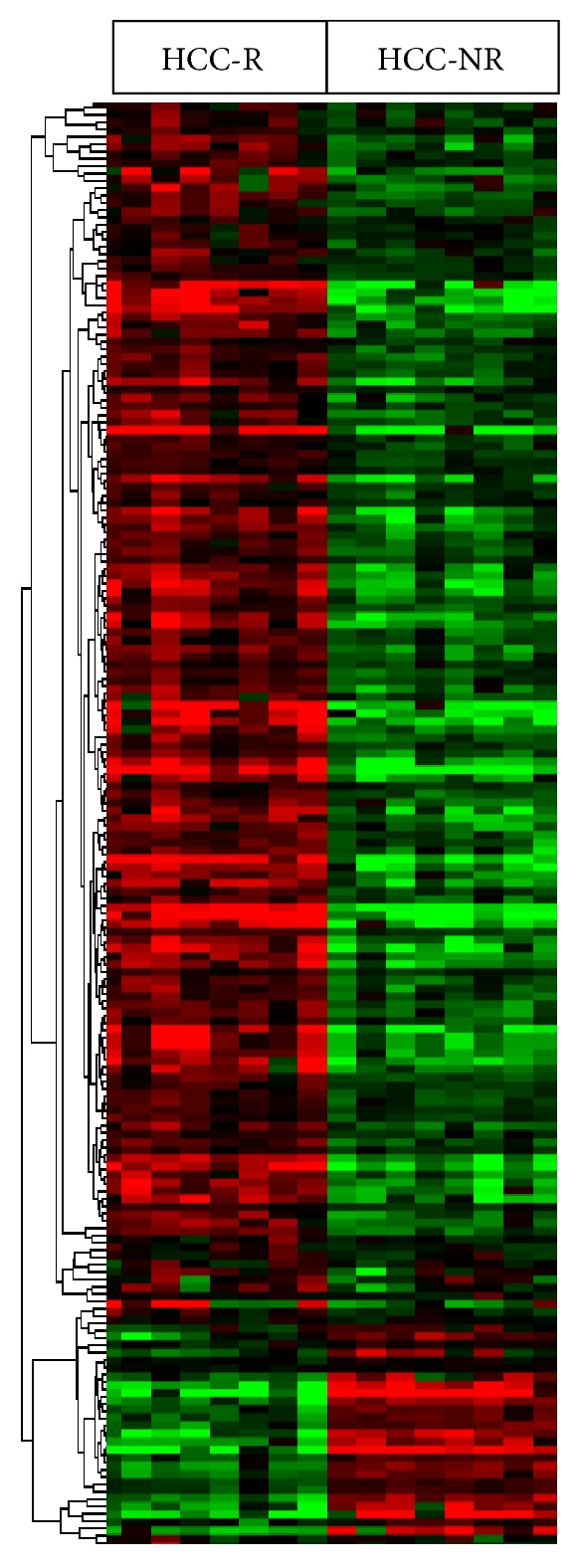
Hierarchical clustering for 194 genes differentially expressed in HCC-R (FDR corrected ≤0.05). For visual comparison, genes differentially expressed in HCC-R and HCC-NR were clustered by the TreeView program. The red color represents genes upregulated, and the green color represents genes downregulated. Saturation was set at ±2-fold change.

The comparative transcriptome analyses aimed at identifying molecular signatures representative of HCC recurrence were carried out as described in Figure 2. Hierarchical clustering by gene expression segregated all HCC-R from HCC-NR. A total of 194 genes were identified to be differentially expressed, with 151 genes upregulated and 43 downregulated in HCC-R (Figure 3).

The biological significance of the altered gene expression pattern described above was investigated by classifying the associated gene within the context of biologically relevant functions using IPA. HCC with recurrence exhibited enrichment of upregulated genes mapping to signaling or disease pathways associated with cell cycle regulators (CDKN2B, E2F2, E2F5, GNL3, HDAC2, MDM2, MYC, and PA2G4), including the genes that encode the proteins that control molecular mechanisms of cancer (FANCD2, FZD3, PLCB1, and PMAIP1). (*P* < 0.0001; Table 2). Gene-encoding proteins implied that nucleo-cytoplasmic transport was also overexpressed (KPNA2, KPNB1, RANBP1, and RCC1). The presence of downregulated pathways reflects the concurrent downregulation of directly related genes categorized in nicotine degradation, complement system, hepatic cholestasis, and catecholamine biosynthesis (*P* < 0.001; Table 2). FXR/RXR activation, which is associated with hepatoprotection [53], was downregulated in recurrent HCC tumors.

### 3.4. Network Analysis Defines Top-Scoring Upregulated Genes Associated with HCC Recurrence

**Figure 4:**
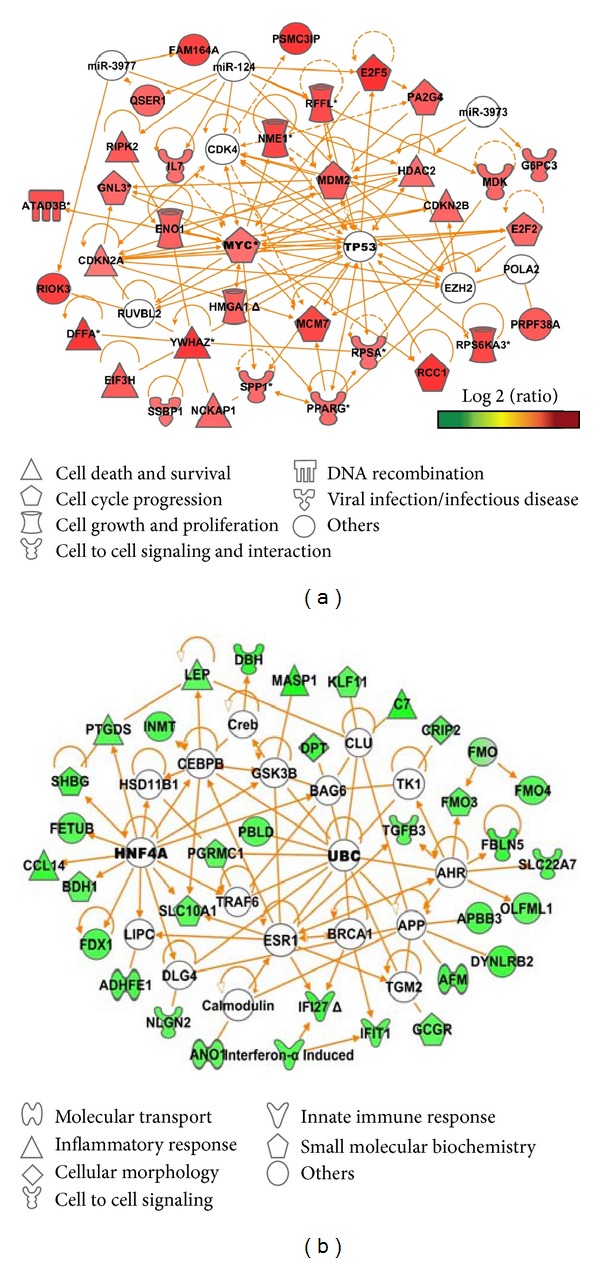
Network analysis of directly related genes which are upregulated and downregulated in HCC-R tumor tissues compared with HCC-NR tumor tissues. (a) The upregulated network includes genes dominated by regulators of cell cycle progression, cell growth and proliferation, DNA recombination, and cell-to-cell signaling genes. These are represented as respective shapes based on functional property in HCC-R tumor tissues. (b) The downregulated network includes genes reflecting the concurrent downregulation of genes related to innate immune response, cell-to-cell signaling, cell morphology, and cellular metabolism genes. These are represented as respective shapes based on their functional properties in HCC-R tumor tissues. The gray outlined nodules without expression are linker genes that are not altered in HCC-R but are statistically enriched for interaction with the altered genes.

Interestingly, when network analysis was performed, which allows exploration of the biological relationship between any two genes, it was seen that the largest number of genes with higher expression in HCC-R tumor tissue was dominated by major transcriptional regulators. These genes are associated with cellular malignancies by controlling cell cycle progression, cell growth and proliferation, cell-to-cell signaling, and cell survival and death. Additionally, a novel set of highly significant genes associated with tumor recurrence was identified (Figure 4(a)).

#### 3.4.1. Activation of the Major Transcriptional Regulators

The expression data revealed an increased (≥3-fold) expression of transcriptional regulators in HCC-R tumor tissues (MYC (3.72), CTNNB1 (3.19), and MDM2 (4.27)). The respective *z*-score for MYC is 2.080 (*z* ≤ 3), as obtained from the IPA transcription factor (TF) analysis, which predicts the activation of TFs based on the expression levels of their known targets. *β*-catenin (CTNNB1), another major transcriptional regulator of specific oncogenes, was also overexpressed in the HCC-R tissue. Many of the downstream targets of the transcription factors MYC and CTNNB1 were upregulated in a fashion consistent with their increased abundance.

#### 3.4.2. Genes Associated with Cellular Malignancies

A number of key genes related to cellular malignancies were upregulated in HCC-R tumors, specifically, HMGA1 (4.16), SPP1 (3.90), GNL3 (3.86), and PPARG (3.84). Two major kinases, including the cyclin-dependent kinase inhibitor 2A (CDKN2A) and cyclin-dependent kinase inhibitor 2B (CDKN2B), showed significantly increased expression (fold changes 3.52 and 3.99, resp.) and are known to be associated with abnormal cell growth. A highly significant up-regulation of a large number of genes (*P* ≤ 0.00001) implicated cell death and survival. The network included genes with fold change ≥4: RIPK2 (4.35) and NCKAP1 (4.02). Additionally, MDK (3.99), NME1 (4.82), and PA2G4 (4.21) showed increased expression in the tumor tissues from the patients with recurrent HCC.

#### 3.4.3. Novel Highly Significant Genes Associated with Tumor Recurrence

A set of genes including PRPF38A, RIOK3, QSER1, PSMC3IP, ATAD3B, MGC12982, and C20ORF27 were significantly overexpressed in HCC-R tumor tissue with fold changes ≥4 (*P* = 0.001 to 0.0001). Most of these genes are known to be involved in various regulatory mechanisms such as cell cycle regulation and DNA replication and recombination mechanism (Table 3); however, the mechanism of action is not well characterized. QSER1, MGC12982 and C20ORF27 are completely uncharacterized. Genes such as MCM7 (4.78), DFFA (5.24), and PRPF38A (4.28) are engaged in gene regulation while E2F5 (6.60), RPS6KA3 (4.77), and YWHAZ (5.18) are associated with cell proliferation. Anti-apoptotic genes such as RFFL (5.32), EIF3H (4.27), and HDAC2 (4.05) were overexpressed. RIOK3 (5.00; *P* ≤ 0.0001) and RCC1 (5.32; *P* ≤ 0.0002) are the top significant genes associated with cytoskeletal architecture and the cellular transport mechanism.

### 3.5. Network Analysis Defines Top-Scoring Downregulated Genes Associated with HCC Recurrence

Underexpressed genes were categorized in regulating innate immune response, cell-to-cell signaling and interaction, and the inflammatory response (Figure 4(b)). Two major transcriptional regulators, the hepatocyte nuclear factor 4, alpha (HNF4A) and ubiquitin C (UBC) genes, had no predictive expression value in HCC-R tumor tissues.

#### 3.5.1. Inactivation of the Major Transcriptional Regulators

The downregulated network includes two major transcriptional regulators, HNF4A and UBC, with no predictive expression values. However, most of the downstream targets of the transcription factors HNF4A and UBC were downregulated in a fashion consistent with their decreased abundance.

#### 3.5.2. Genes Associated with Innate Immunity, Cell-to-Cell Signaling and Interaction

The genes that displayed the most dramatic downregulation were MASP1 (−5.11), C7 (−5.06), DBH (−4.66), and FBLN5 (−4.48). However, such genes as IFI27 (−4.38), SLC22A7 (−3.97), IFIT1 (−3.91), TGFB3 (−3.71), and IFN-*α* induced (−3.72) also had stronger suppression at the transcript level in HCC-R tumor tissues.

#### 3.5.3. Genes Associated with Inflammatory Response

The inflammatory response-related genes (CCL14 (−4.52), LEP (−3.73) and PTGDS (−3.84)) showed decreased expression in the recurrence tumor tissues.

#### 3.5.4. Other Significant Genes

Several genes, SLC10A1, GCGR, FMO3, and INMT, were previously known to be downregulated in HCV-induced HCC [28, 54]. These genes showed similar expression changes with the fold change ≥3.5. Additionally, a set of genes, including AFM, DYNLRB2, FDX1, and SHBG, were significantly downregulated in HCC-R tumor tissues with fold changes ≥4 involved in various small molecule biochemistry and molecular transport mechanisms. These genes were previously known to be suppressed in HCC [55–57].

### 3.6. Validation of Gene Expression in HCC-R and HCC-NR Tumor Tissues

**Figure 5:**
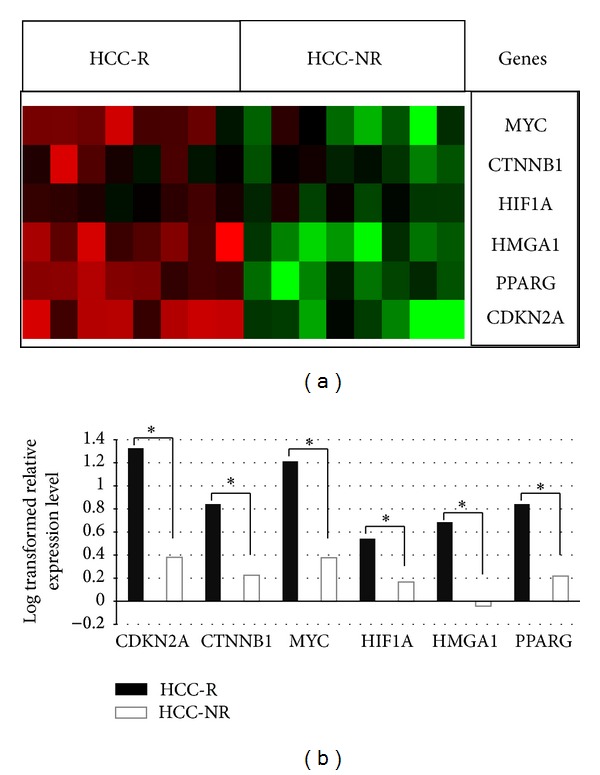
Validation of microarray data by qPCR. The *P* values were calculated with Student's *t*-test (*P < 0.05).

**Figure 6:**
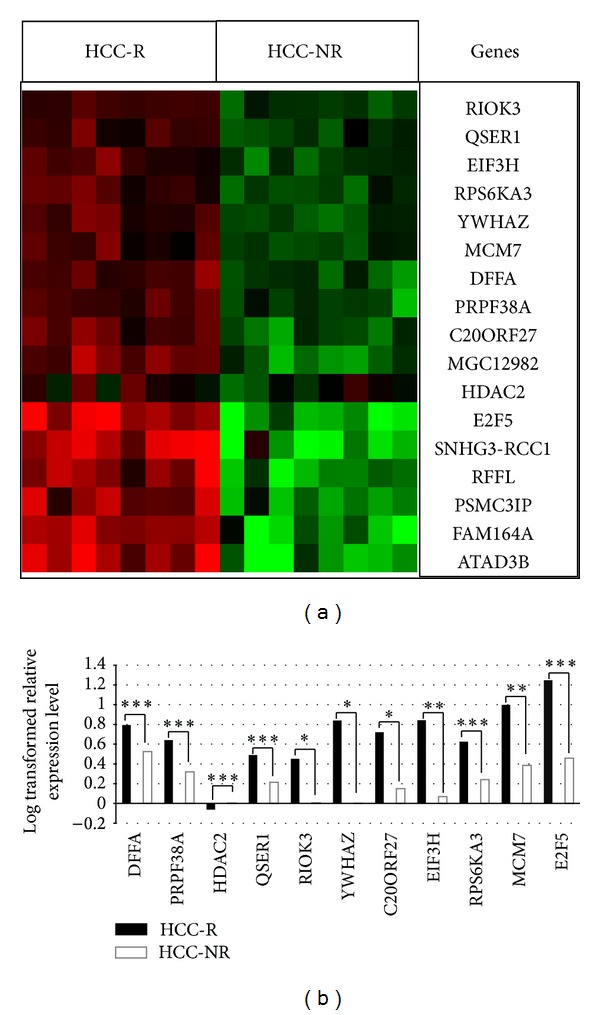
Validation of microarray data by qPCR. The *P* values were calculated with Student's *t*-test (*** < 0.05; ** < 0.07; * < 0.25).

Total RNA from the same two groups of patients, the HCC-R group and the HCC-NR group, was used for qPCR analysis to validate microarray expression data. The following genes were selected for validation from a set of novel highly significant upregulated genes from our expression dataset (Table 3): DFFA, RIOK3, E2F5, EIF3H, YWHAZ, QSER1, RPS6KA3, PRPF38A, MCM7, and C20ORF27. Additionally, we selected a few genes (CTNNB1, PPARG, HIF1A, HMGA1, MYC, and CDKN2A with *P* values <0.05; Table 4) that are a hallmark of HCC in the literature [47, 49, 50, 58, 59]. The expression levels determined by qPCR were comparable to the microarray data in HCC-R versus HCC-NR tissue (Figures 5 and 6).

## 4. Discussion 

Recurrent HCC following surgical treatment or liver transplantation continues to be a serious health problem [60]. New therapeutic methods need to be developed. The aim of genomic analysis is to enable development of these new treatment options. Presently, the molecular mechanisms involved in HCV-infected individuals who develop HCC recurrence are largely unknown.

To find these molecular mechanisms, several studies using many samples will need to be conducted. This present study has revealed FFPE tissue to be a good source of such study material; the use of FFPE tissue will greatly expand the number of samples available for study.

The present study revealed both known and previously unknown molecular patterns associated with HCC recurrence; however, this is a proof of concept study on a small subset of HCC patients. All patients were chronically infected with HCV, most were Caucasian, and the average age was 55 years. Tumor characteristics varied in terms of histology, vascular invasion, tumor grade, and number and size of tumors. The small sample size and wide variety of tumor characteristics do not allow for any strong conclusions to be drawn from this study; the highly differentiated levels of gene expression are interesting to review, however.

In this study, the use of FFPE-stored tissues for whole transcriptome analysis provided both previously known and novel insights into HCV-associated HCC with or without recurrence. A set of 194 genes differentially expressed was observed in two groups of patients with HCV and either experiencing or not experiencing HCC recurrence. The present study identified and quantified 6 genes that were highly overexpressed in our HCC-R samples (CTNNB1, PPARG, HIF1A, HMGA1, MYC, and CDKN2A) that other investigators have determined to be hallmarks of HCC [46, 47, 49, 50]. Additionally, the present study identified, for the first time, a set of highly significant upregulated genes in the HCC-R tumor tissues. These genes are involved with gene regulation (DFFA, MCM7, and PRPF38A), cell proliferation (E2F5, RPS6KA3, and YWHAZ) cytoskeletal architecture (RIOK3), anti-apoptosis (EIF3H, RFFL, and HDAC2) and uncharacterized functions (QSER1, MGC12982, and C20ORF27). The increased expression of anti-apoptotic genes, including EIF3H, RFFL and HDAC2, may favorably assist uncontrolled cell growth and proliferation to enhance tumor growth in patients with HCC-R. Previous studies have shown that RFFL and HDAC2 are known to also promote tumor formation by inhibiting the apoptosis process [26, 27, 61], whereas EIF3H is known to increase cell proliferation, growth and survival and inhibit the apoptosis process [44]. We observed an increased expression of the YWHAZ gene, which is known as a potential metastasis factor with the antiapoptotic property of promoting cellular malignancy [38, 62]. Overall data suggests that HCC recurrence is associated with increased apoptosis inhibition, and uncontrolled cell growth leads to a tumor metastasis microenvironment that favors tumor recurrence in patients with HCC-R. Of note is that a major hallmark of an aggressive HCC is its ability to metastasize [63]; the recurrence of HCC supports the metastatic phenomenon. The observed increased expression of RCC1 is one of the most important members of the RAN signaling pathway, which is involved in the nucleocytoplasmic transport of macromolecules [64, 65]. Recent findings have shown that silencing RAN expression could induce more apoptosis in cancer cells, and therefore is a promising cancer therapeutic target [66]. This suggests a novel link between the elevated RAN signaling pathway in HCC recurrence and a potentially important role for nucleo-cytoplasmic transport mechanisms of RCC1 during HCC progression. The increased expression of RIOK3 is known to alter the cytoskeletal architecture, as well as promoting pancreatic ductal cell migration and invasion [30]. The increased expression of RIOK3 has been observed in metastatic head and neck cancers compared with nonrecurrent tumors [67]. These observations raise an interesting prospect that similarly, increased expression of RIOK3 may contribute to cytoskeletal architecture alteration to influence cell migration and tumor invasion in HCC patients having tumor recurrence. Thus, the findings of the present study further point toward new avenues of research aimed at evaluating the impact of anti-apoptosis, cytoskeletal architecture alteration, and RAN signaling on HCC recurrence.

A limitation of this study was the use of only 50% of the FFPE tissue with microarray analysis and the low number of tissues used for the final analysis. Further investigation with larger cohort is warranted. Comparing specific subgroups of different liver diseases, races/ethnicities, ages, and tumor characteristics could reveal clinical implications that could potentially aid in patient selection for liver transplantation. Future studies with recent advanced technology such Next Generation RNA Sequencing (RNA-seq) might offer greater potential for the use of FFPE samples, with a tremendous increase in the number of samples to study. RNA-seq, a recently developed approach to transcriptome profiling that uses deep-sequencing technologies [68], could offer a greater opportunity to use FFPE samples [8, 69] to bring gene expression results into the clinical treatment of HCC.

In conclusion, this pilot expression profiling study using FFPE tissue has shown that stored FFPE tissue is a vital resource and has identified molecular patterns for HCC-R tumor tissue consistent with prior studies. We also identified a set of genes not previously reported to be associated with HCC-R. All of these genes may be potential targets for future therapeutic interventions.

## Figures and Tables

**Table 1:** Patient characteristics: clinical characteristics of the 16 patients from tumor recurrence and tumor nonrecurrence groups (mean ± SD or proportion).

Study group	HCC-R group	HCC-NR group	*P*-value
Number	8	8	
Age, years	57.7 ± 5.1	52.1 ± 7.4	0.1
Male gender	100%	100%	1
Race			
Caucasian	75%	100%	0.2
Asian	12.5%	0%	
Alaska native	12.5%	0%	
BMI*	28.5 ± 5	27.3 ± 2.3	0.5
AFP level, mg/dL**	2268 ± 2837	138.1 ± 280	0.07
Cirrhosis, postnecrotic-type C	100%	100%	1
Prior HCV treatment	75%	50%	0.3
Prior HCC treatment	37.5%	0%	0.2
Hours to process***	39 ± 24.9	17.9 ± 12.7	0.06
Largest diameter Tumor, cm	4.2 ± 2.4	2.8 ± .7	0.1
Number of tumors			
1	25%	50%	0.2
2	25%	12.5%	
(3–5)	0%	37.5%	
≥6	50%	0.0%	
Tumor in both lobes	37.5%	12.5%	0.6
Histology			
Poorly differentiated	75%	0%	<0.01
Moderately differentiated	25%	75%	
Well differentiated	0%	25%	
Macroinvasion	50%	0%	0.08

*Body mass index; **alpha-fetoprotein level.

***Time specimens removed in surgical procedure until being fixed in formalin.

**Table 2:** Top canonical pathways: significant molecular pathways regulated in HCC-R.

Up-regulated in HCC-R	*P* value	Genes
Cell cycle: G1/S checkpoint regulation	9.16*E* − 09	CDKN2B, E2F2, E2F5, GNL3, HDAC2, MDM2, MYC, PA2G4
RAN signaling	4.92*E* − 06	KPNA2, KPNB1, RANBP1, RCC1
Chronic myeloid leukemia signaling	4.89*E* − 05	E2F2, E2F5, HDAC2, MDM2, MYC, PA2G4
Molecular mechanisms of cancer	1.35*E* − 04	CDKN2B, E2F2, E2F5, FANCD2, FZD3, MDM2, MYC, PA2G4, PLCB1, PMAIP1
Cyclins and cell cycle regulation	2.22*E* − 04	CDKN2B, E2F2, E2F5, HDAC2, PA2G4

Downregulated in HCC-R	*P*-value	Genes

Nicotine degradation II	3.41*E* − 04	FMO3, FMO4, INMT
FXR/RXR activation	1.05*E* − 03	FETUB, SLC10A1, SLC22A7
Complement system	2.69*E* − 03	C7, MASP1
Hepatic cholestasis	4.14*E* − 03	GCGR, SLC10A1, SLC22A7
Catecholamine biosynthesis	9.25*E* − 03	DBH

**Table 3:** Highly significant upregulated genes in HCC-R tumors with known and unknown functions (references are based on PUBMED search).

ILMN Gene ID no.	Genes	*P *	FC	Known cellular function (Ref.)	Role in HCC (Ref.)	HCC-R secondary to HCV (Ref.)
1667213	DNA fragmentation factor, 45 kDa, alpha polypeptide (**DFFA**)	0.0011	5.24	(1) Tumor suppressor gene [22]. (2) Protein encoded by DFFA gene known as inhibitor of caspase-activated DNase (ICAD), involved in DNA fragmentation during apoptosis [23].	Unknown	Unknown

1664111	Ring finger and FYVE-like domain containing E3 ubiquitin protein ligase (**RFFL**)	0.0005	4.72	(1) RFFL is a member of caspase 8/10-associated RING proteins (CARPs) are a recently described family of protein ubiquitin ligases that interact with and negatively regulate death receptor-mediated apoptosis [24].	Unknown	Unknown

1675626	PRP38 pre-mRNA processing factor 38 (yeast) domain containing (**PRPF38A**)	0.0005	4.28	(1) PRP38 pre-mRNA processing factor 38 (yeast) domain containing A protein involved in pre-mRNA splicing [25].	Unknown	Unknown

1767747	Histone deacetylase 2 (**HDAC2**)		4.05	(1) This gene product belongs to the histone deacetylase family known to play an important role in transcriptional regulation, cell cycle progression and developmental events. (2) Role in antiapoptosis in tumor cells [26].	[27]	Unknown

2147863	Glutamine and serine rich 1 (**QSER1**)		3.98	Unknown	Unknown	[28]

2354211	Regulator of chromosome condensation 1 (**RCC1**)	0.0002	5.32	(1) Regulator of chromosomal condensation [29].	Unknown	Unknown

2404135	RIO kinase 3 (yeast) (**RIOK3**)	<0.0001	5.00	(1) The specific function of this gene has not yet been determined (NCBI). (2) Recent study identified Rio kinase 3 (RIOK3) as an amplified gene that alters cytoskeletal architecture as well as promotes pancreatic ductal cell migration and invasion [30].	Unknown	

1770822	Ribosomal protein S6 kinase, 90 kDa, polypeptide 3 (**RPS6KA3**)	0.0001	4.77	(1) Known to regulating cell growth and differentiation [31]. (2) Known to play a role in the cellular antiviral response [32].	[33]	Unknown

1663195	Minichromosome maintenance complex component 7 (**MCM7**)	0.0031	4.78	(1) Respective protein involved in regulating DNA replication [34]. (2) MCM7 overexpression and amplification and several human malignancies [35].	[36]	Unknown

1801928	Tyrosine 3-monooxygenase/tryptophan 5-monooxygenase activation protein, zeta polypeptide (**YWHAZ**)	0.0007	5.18	(1) The encoded protein interacts with IRS1 protein, suggesting a role in regulating insulin sensitivity [37]. (2) Known to promote malignancies in many cell types [38].	[39]	Unknown

2057981	Zinc finger, C2HC-type containing 1A (**FAM164A**)	0.0008	4.90	Unknown	Unknown	Unknown

1782551	E2F transcription factor 5, p130-binding (**E2F5**)	<0.0001	6.60	(1) A transcription factor regulates cell proliferation and differentiation [40]. (2) Over-expressed in several human malignancies [41].	[37]	Unknown

3307266	PSMC3 interacting protein (**PSMC3IP**)	0.0006	5.23	(1) This gene encodes a protein that functions in meiotic recombination [42].	Unknown	Unknown

2131936	ATPase family, AAA domain containing 3B (**ATAD3B**)	0.001	4.55	(1) ATAD3B is mitochondrial membrane proteins that contribute to the stabilization of large mitochondrial DNA (mtDNA) protein complexes called nucleoids [43].	Unknown	Unknown

1683660	Eukaryotic translation initiation factor 3, subunit H (**EIF3H**)	0.0016	4.27	(1) At the cellular level, EIF3H overexpression increases proliferation, growth and survival [44]. (2) Inhibit induction of apoptosis [44].	[45]	Unknown

3309468	Hypothetical protein MGC12982 (**MGC12982**)	0.0006	4.09	Unknown	Unknown	Unknown

1697363	Chromosome 20 open reading frame 27 (**C20ORF27**)	0.0003	4.15	Unknown	Unknown	Unknown

*P* = *P* value; FC = fold change.

**Table 4:** Gene list known to be deregulated in HCC selected from our microarray data for cross validation as positive control (references are based on PUBMED search).

Genes	FC	Role in HCC (Ref.)
v-myc myelocytomatosis viral oncogene homolog (avian) (**MYC**)	3.72	[46]
Catenin (cadherin-associated protein), beta 1, 88 kDa (**CTNNB1**)	3.19	[47]
Hypoxia inducible factor 1, alpha subunit (basic helix-loop-helix transcription factor) (**HIF1A**)	3.06	[48]
High mobility group AT-hook 1 (**HMGA1**)	4.16	[49]
Peroxisome proliferator-activated receptor gamma (**PPARG**)	3.84	[50]
Cyclin-dependent kinase inhibitor 2A (**CDKN2A**)	4.52	[51, 52]

FC = fold change (based on our expression data).

## Abbreviations

HCV: Hepatitis C virus
HCC: Hepatocellular carcinoma
BMI: Body mass index
FFPE: Formalin-fixed paraffin-embedded
IRB: Institutional Review Board
AFP: Alpha-fetoprotein
PCRS: Predicting cancer recurrence score
DASL: cDNA-mediated annealing, selection, extension, and ligation
QC: Quality control
IPA: Ingenuity pathway analysis
PCA: Principal component analysis
ATAD3A/ATAD3B: ATPase family, AAA domain containing 3A
DASL: cDNA-mediated annealing, selection, extension, and ligation
CDK4: Cyclin-dependent kinase 4
CDKN2A: Cyclin-dependent kinase inhibitor 2A
CDKN2B: Cyclin-dependent kinase inhibitor 2B (p15, inhibits CDK4)
DFFA: DNA fragmentation factor, 45 kDa, alpha polypeptide
E2F2: E2F transcription factor 2
E2F5: E2F transcription factor 5, p130-binding
EIF3H: Eukaryotic translation initiation factor 3, subunit H
ENO1: Enolase 1, (alpha)
EZH2: Enhancer of zeste homolog 2 (*Drosophila*)
FDR: False discovery rate
G6PC3: Glucose 6 phosphatase, catalytic, 3
GNL3: Guanine nucleotide binding protein-like 3 (nucleolar)
HCC-NR: Hepatocellular carcinoma nonrecurrence
HCC-R: Hepatocellular carcinoma recurrence
HDAC2: Histone deacetylase 2
IL7: Interleukin 7
LT: Liver transplantation
MCM7: Minichromosome maintenance complex component 7
MDK: Midkine (neurite growth-promoting factor 2)
MDM2: Mdm2, p53 E3 ubiquitin protein ligase homolog (mouse)
MYC: v-myc myelocytomatosis viral oncogene homolog (avian)
NCKAP1: NCK-associated protein 1
NME1: NME/NM23 nucleoside diphosphate kinase 1
PA2G4: Proliferation-associated 2G4, 38 kDa
POLA2: Polymerase (DNA directed), alpha 2, accessory subunit
PPARG: Peroxisome proliferator-activated receptor gamma
PRPF38A: PRP38 pre-mRNA processing factor 38 (yeast) domain containing A
PSMC3IP: PSMC3 interacting protein
qPCR: Quantitative real-time polymerase chain reaction
QSER1: Glutamine and serine rich 1
RCC1: Regulator of chromosome condensation 1
RFFL: Ring finger and FYVE-like domain containing E3 ubiquitin protein ligase
RIOK3: RIO kinase 3 (yeast)
RIPK2: Receptor-interacting serine-threonine kinase 2
RPS6KA3: Ribosomal protein S6 kinase, 90 kDa, polypeptide 3
RPSA: Ribosomal protein SA
RUVBL2: RuvB-like 2 (*E. coli*)
SPP1: Secreted phosphoprotein 1
SSBP1: Single-stranded DNA binding protein 1, mitochondrial
TP53: Tumor protein p53
YWHAZ: Tyrosine 3-monooxygenase/tryptophan 5-monooxygenase activation protein, zeta polypeptide
ZC2HC1A: Zinc finger, C2HC-type containing 1A
ADHFE1: Alcohol dehydrogenase, iron containing, 1
AFM: Afamin
AHR: Aryl hydrocarbon receptor
ANO1: Anoctamin 1, calcium activated chloride channel
APBB3: Amyloid beta (A4) precursor protein-binding, family B, member 3
APP: Amyloid beta (A4) precursor protein
BAG6: BCL2-associated athanogene 6
BDH1: 3-Hydroxybutyrate dehydrogenase, type 1
BRCA1: Breast cancer 1, early onset
C7: Complement component 7
CCL14: Chemokine (C-C motif) ligand 14
CEBPB: CCAAT/enhancer binding protein (C/EBP), beta
CLU: Clusterin
CRIP2: Cysteine-rich protein 2
DBH: Dopamine beta-hydroxylase (dopamine beta-monooxygenase)
DLG4: Discs, large homolog 4 (Drosophila)
DPT: Dermatopontin
DYNLRB2: Dynein, light chain, roadblock-type 2
ESR1: Estrogen receptor 1
FBLN5: Fibulin 5
FDX1: Ferredoxin 1
FETUB: Fetuin B
FMO3: Flavin containing monooxygenase 3
FMO4: Flavin containing monooxygenase 4
GCGR: Glucagon receptor
GSK3B: Glycogen synthase kinase 3 beta
HNF4A: Hepatocyte nuclear factor 4, alpha
HSD11B1: Hydroxysteroid (11-beta) dehydrogenase 1
IFIT1: Interferon-induced protein with tetratricopeptide repeats 1
INMT: Indolethylamine N-methyltransferase
KLF11: Kruppel-like factor 11
LEP: Leptin
LIPC: Lipase, hepatic
MASP1: Mannan-binding lectin serine peptidase 1 (C4/C2 activating component of Ra-reactive factor)
NLGN2: Neuroligin 2
OLFML1: Olfactomedin-like 1
PBLD: Phenazine biosynthesis-like protein domain containing
PGRMC1: Progesterone receptor membrane component 1
PTGDS: Prostaglandin D2 synthase 21 kDa (brain)
SHBG: Sex hormone-binding globulin
SLC10A1: Solute carrier family 10 (sodium/bile acid cotransporter family), member 1
SLC22A7: Solute carrier family 22 (organic anion transporter), member 7
TGFB3: Transforming growth factor, beta 3
TGM2: Transglutaminase 2 (C polypeptide, protein-glutamine-gamma-glutamyltransferase)
TK1: Thymidine kinase 1, soluble
TRAF6: TNF receptor-associated factor 6, E3 ubiquitin protein ligase
UBC: Ubiquitin C.
